# A new link between insulinoma and congenital glucose-galactose malabsorption

**DOI:** 10.1530/EO-25-0030

**Published:** 2025-07-11

**Authors:** Antonio Prinzi, Jelka Kuiper, Adorée M van der Wiel, Marie-Louise F van Velthuysen, Remco T P van Cruchten, Lodewijk A A Brosens, Wouter W de Herder, Johannes Hofland

**Affiliations:** ^1^ Endocrinology Unit, Department of Clinical and Experimental Medicine, University of Catania, Garibaldi-Nesima Medical Center, Catania, Italy; ^2^ Department of Internal Medicine, Section of Endocrinology, ENETS Center of Excellence, Erasmus MC Cancer Institute, Rotterdam, The Netherlands; ^3^ Department of Internal Medicine, Section of Dietetics, Erasmus MC, Rotterdam, The Netherlands; ^4^ Department of Pathology, Erasmus MC, Rotterdam, The Netherlands; ^5^ Department of Pathology, Radboud University Medical Center, Nijmegen, The Netherlands; ^6^ Department of Pathology, University Medical Center Utrecht, Utrecht University, Utrecht, The Netherlands

**Keywords:** congenital glucose galactose malabsorption, insulinoma, neuroendocrine tumor, SGLT-1

## Abstract

Congenital glucose-galactose malabsorption (CGGM) is a rare autosomal recessive disorder caused by a biallelic mutation of solute carrier family 5 member 1 (*SLC5A1*), encoding the sodium-dependent glucose transport-1 (SGLT-1) protein. Patients with CGGM present with neonatal-onset osmotic diarrhea due to impaired intestinal uptake of glucose. Here, we report a case of a 41-year-old female with CGGM who was referred to our clinic for symptoms of hypoglycemia, with the final diagnosis of an insulinoma, which was successfully resected. Whole genome sequencing of tumor DNA revealed chromosomal aberrations, without the presence of driver mutations. Given the unknown long-term sequelae of SGLT-1 loss of function in adulthood, this first case of insulinoma in a CGGM patient potentially uncovers a new phenotype resulting from decades of imbalance in glucose homeostasis. We hypothesize that SGLT-1 might play a role in the plasticity of pancreatic β-cells and suggest mechanisms through which CGGM patients could potentially have a higher risk of developing insulinoma in adulthood.

**Learning points:**

- This is the first documented case of insulinoma in a patient with CGGM, suggesting a potential new link between glucose absorption disorders and pancreatic neuroendocrine tumors.
- The loss-of-function mutation in SGLT-1 due to an SLC5A1 gene mutation may impact pancreatic β-cell plasticity, potentially contributing to insulinoma development through altered glucose homeostasis.
- The patient’s long-term high-fat, low-carbohydrate diet may have played a role in β-cell stimulation via increased levels of GLP-1 and GIP, both of which promote β-cell proliferation and survival.

## Background

Insulinomas are rare functioning neuroendocrine tumors (NETs) of the pancreas, with an estimated incidence of 1–4 cases per million per year (Hofland *et al.* 2024). Approximately 10–15% of insulinomas display aggressive behavior and are characterized by metastatic potential, while 85–90% can be considered indolent (Rindi *et al.* 2022, Hackeng *et al.* 2023). Indolent insulinomas usually present as small lesions (<3 cm), become symptomatic with hypoglycemia early, and exhibit a genetic and epigenetic signature close to their presumed cell of origin, the β-cell, due to expression of pancreatic and duodenal homeobox 1 (PDX1) and glucagon-like peptide-1 receptor (GLP-1R) (Di Domenico *et al.* 2020, Hackeng *et al.* 2020, 2023, Rindi *et al.* 2022). Insulinomas may occur sporadically or, in a minority of cases, as part of multiple endocrine neoplasia type 1 (MEN1) syndrome, tuberous sclerosis, or neurofibromatosis type 1 (De Herder 2004, Hofland *et al.* 2023). In the present study, we describe a case of insulinoma arising in a patient with congenital glucose-galactose malabsorption (CGGM). CGGM is an ultra-rare autosomal recessive disorder caused by a defect in glucose and galactose transport across the small intestine, caused by a biallelic mutation in the solute carrier family 5 member 1 gene (*SLC5A1*), leading to loss of function of the sodium-dependent glucose transport-1 protein (SGLT-1) (Wang *et al.* 2020). This defect leads to lack of intestinal absorption of glucose, galactose, and sodium, causing hyperosmotic diarrhea and dehydration at a neonatal age (Wright *et al.* 2003). Case reports of this disease have been described up to the age of 26 years and have presented no other phenotype than osmotic diarrhea (Xin & Wang 2011). Treatment of CGGM varies according to age. In infancy, the primary source of nutrition is a fructose-based formula, either a carbohydrate-free formula with added fructose or a pre-made formula containing only fructose as a source of carbohydrate. Typically, by 4 to 6 months of age, low-carbohydrate foods such as fruits and vegetables can be introduced. Over time, additional carbohydrates may be gradually included, with some studies indicating improved tolerance to glucose and galactose as the child grows. While the nutritional management of CGGM during infancy is well documented, there is limited information on long-term follow-up (Abad-Sinden *et al.* 1997, Scanlan *et al.* 2012, Saadah *et al.* 2014).

## Case presentation

A 41-year-old female was referred to our clinic because of the suspicion of hypoglycemia following multiple episodes of hypoglycemia-related symptoms, predominantly occurring during fasting. The patient was diagnosed with CGGM at the age of 4 weeks and was treated with a fructose-based diet. Her case was reported in 1981 by Douwes *et al.* (1981). DNA analysis confirmed a biallelic *SLC5A1* [NM_000343.4] c.884A>G, p.(Gln295Arg) mutation. Since adolescence, she switched to a high-fat diet, as only excessive glucose intake was associated with diarrhea. At current presentation, she reported symptoms of nightly neuroglycopenia, including limb paralysis and confusion, and rare episodes of fainting during the past 2 years. These symptoms occurred during hypoglycemia and dissipated after ingestion of carbohydrates. In addition, the patient reported a weight gain of 10 kg. On examination, her body mass index was 25.8 kg/m^2^ (weight: 78 kg; height: 174 cm). Bioelectrical impedance analysis revealed a fat mass of 30.2 kg (corresponding to the 90th–95th percentile) and a fat-free mass of 47.8 kg, resulting in a body fat percentage of 38.7%. In our clinic, a prolonged fasting test was performed and interrupted after 19 h because of the presence of hypoglycemic symptoms. Hormonal levels at the time of hypoglycemia were compatible with endogenous hyperinsulinemic hypoglycemia (Table 1) (Hofland *et al.* 2023).

**Table 1:** Biochemical evaluations during fasting test.

Parameters	Value	Cut-offs for endogenous hyperinsulinemic hypoglycemia*
Plasma glucose (mmol/L)	1.89	<2.5
Plasma insulin (mIU/L)	61.8	≥20.8
C-peptide (nmol/L)	0.87	≥0.2
Pro-insulin (pmol/L)	>100	≥5

* Hofland *et al.* (2023)

## Investigation

Because of the suspicion of insulinoma, computed tomography (CT) and magnetic resonance imaging of the abdomen, ^68^Ga-DOTATATE positron emission tomography (PET)/CT, and ^68^Ga-Exendin-4 PET/CT were sequentially performed, but no suspicious lesions were visualized in the pancreas. Therefore, the patient was submitted to endoscopic pancreatic ultrasonography and selective arterial calcium stimulation (SACST) with hepatic venous sampling, which were suggestive of insulinoma in the head of the pancreas.

## Treatment

Following diagnosis, a pancreaticoduodenectomy was performed. Histopathological examination showed a well-differentiated neuroendocrine tumor of 1.9 cm in size (pT1N0). On immunohistochemistry, the tumor stained positive for insulin, CK18, SSTR2A, chromogranin, and synaptophysin. The Ki-67 was 2%, and the mitotic count was 2/10 HPF, compatible with a grade 1 neuroendocrine tumor. The adjacent pancreatic islets appeared normal. The tumor was positive for PDX1 and negative for ARX, consistent with a NET of β-cell origin. There was no loss of menin, ATRX, or DAXX staining.

## Outcome and follow-up

**Figure 1:**
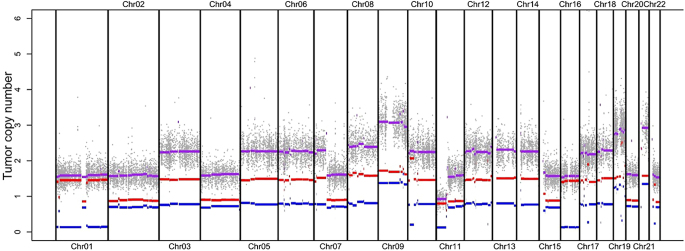
Copy number profile of the insulinoma. Red and blue lines indicate the copy numbers of the two alleles; their sum is represented in purple. Note that, for visualization purposes, an artificial distance was included for overlapping lines of equal copy number. Plot generated with PureCN.

After removal of the insulinoma, the patient no longer experienced hypoglycemia and had no biochemical or radiological recurrence during 5 years of follow-up. Mutational analysis of tumor tissue did not reveal any pathogenic mutations for *MEN1, RB1, TP53, TSC1, TSC2, YY1, ATRX, DAXX,* and *RHEB* in the insulinoma. Large-scale copy number variations (CNVs) in the form of copy-neutral loss of heterozygosity (LOH) were detected for the complete chromosomes 1 and 16, while copy-loss LOH for chromosome 11p (Fig. 1). In addition, chromosomal gains were observed for chromosomes 3, 5, 6, 7p, 8, 10, 12, 13, 14, 17, and 18 (3n), and for chromosomes 9, 19, and 21 (4n). No significant alterations were observed for chromosomes 2, 4, 7q, 11q, 15, 20, 22, and X.

### DNA analysis

For the germline SGLT-1 mutation, the 15 exons of the *SLC5A1* gene and 527 base pairs of the promoter region were analyzed for single-stranded conformational polymorphisms (SSCPs), and subsequently sequenced via dideoxy-sequencing (Douwes *et al.* 1981). Tumor DNA of the insulinoma was isolated from FFPE tissue to investigate somatic mutations and CNVs. Whole exome sequencing was performed via enrichment with Twist Comprehensive Exome 2.0 probes and sequencing on an Illumina NovaSeq6000. Data analysis was performed with an in-house bioinformatic pipeline relying on bwa-mem2 for alignment, Mutect2 for variant calling, and PureCN for copy-number analysis (Riester *et al.* 2016, Benjamin *et al.* 2019, Vasimuddin *et al.* 2019).

### Pathological examination

Immunohistochemical staining was conducted on the tumor sample for insulin (Klinipath MU029-UC, The Netherlands), CK18 (Cell Marque 760-4344, USA), and SSTR2A (Clinisciences NB-49-015, France); chromogranin A (Roche 760-2519, Switzerland); synaptophysin (Roche 790-4407); Ki-67 (Roche 790-4286); PDX1 (Abcam ab134150, USA); ARX (Millipore MABN102, Germany); ATRX (Sigma HPA001906, Germany); DAXX (Merck HPA008736, Germany); and menin (Bethyl Laboratories IHC-00572, USA).

## Discussion

We report a case of CGGM with a biochemically and histologically confirmed insulinoma. CGGM constitutes an ultra-rare autosomal recessive disorder caused by a defect in glucose and galactose transport across the small intestine, due to biallelic mutation in *SLC5A1*, which is located on chromosome 22q13.1 (Wang *et al.* 2020). This mutation causes a defect in SGLT-1 function in the brush-border membrane of the intestine that leads to malabsorption of glucose, galactose, and sodium. The lack of solute uptake subsequently leads to osmotic diarrhea, dehydration, abdominal distension, vomiting, failure to thrive, and weight loss in newborns. Treatment of CGGM is based on a diet free of glucose, galactose, and lactose (Wright *et al.* 2003, Alruwaili & Alshdayed 2023). This dietary restriction leads some individuals to adopt a high-fat and high-protein diet (Wright *et al.* 2003, Anderson *et al.* 2017). An observational study confirmed that patients with CGGM followed a high-fat and high-protein/low-carbohydrate diet, and that fructose was the major source of carbohydrate. Similar to what was observed in our case, patients tended to consume more glucose at increasing age (Chan *et al.* 2021).

Several studies have shown that SGLT-1 is not only expressed in the small intestine but also in various other tissues, such as the heart, liver, skeletal muscle, lung, trachea, gallbladder, colon, rectum, brain, blood vessels, uterus, breast, testis, and pancreatic islets (Koepsell 2017). Within the pancreatic islets, SGLT-1 mRNA and protein expression was observed primarily within the α-cell, while its expression in β-cells was lower (Bonner *et al.* 2015, Berger & Zdzieblo 2020). Given the underlying gene defect and a lack of pancreas neuroendocrine tumor-associated driver mutations, either endocrine or intrinsic islet cell mechanisms could be hypothesized to cause insulinoma formation in this case. The observed CNV profile was concordant with what Hong *et al.* (2020) describe for the ‘insulinoma amplification subtype’. These are described to often lack known driver mutations, as in the case described here. Despite the CNV profile showing chromosomal copy number alteration, a causative association between an *SLC5A1* mutation and formation of β-cell-derived tumors could be supported by two potential pathophysiological mechanisms. A potential stimulatory effect on β-cell mass could be accomplished through the effects of the long-term diet on glucagon-like peptide 1 (GLP-1) and glucose-dependent insulinotropic peptide (GIP) metabolism. A previous study in *Sglt1* knockout (*Sglt1^−/−^*) mice showed that these mice displayed increased circulating levels of GLP-1 and GIP after ingestion of fatty acids, compared to wild-type (Gorboulev *et al.* 2012). Similarly, another study reported that *Sglt1^−/−^* mice fed with a fat-enriched and glucose-deprived diet showed an acute and sustained GLP-1 secretion after oral glucose tolerance test (Mühlemann *et al.* 2018). Pharmacological inhibition of SGLT-1 in rats was also associated with increased plasma GLP-1 levels (Io *et al.* 2019). In humans, ingestion of dietary fat stimulated GLP-1 and GIP release, which might potentially be augmented in CGGM cases (Elliott *et al.* 1993, Herrmann *et al.* 1995). GLP-1 and GIP have been reported to increase cell proliferation and inhibit apoptosis in pancreatic endocrine β-cells, thereby increasing β-cell mass, through the upregulation of IGF-1R and IGF-2 (Drucker 2003, Yusta *et al.* 2006, Baggio & Drucker 2007, Fujimoto & Polonsky 2009, Cornu *et al.* 2010, Ma *et al.* 2014, Zheng *et al.* 2022). It is possible that a diet particularly rich in fat, through the overproduction of GLP-1 and GIP, may play a role in the development of insulinoma because the patient had largely disregarded the diet since puberty by consuming mainly high-fat foods. However, histologically, there were no signs of β-cell hyperplasia in the pancreatic parenchyma surrounding the insulinoma in our patient.

The second hypothesis concerns a local effect of SGLT-1 deficiency within the pancreatic islets. A previous study showed that *Sglt1^−/−^* mice exhibited an aberrant pancreatic islet cytomorphology characterized by enlarged islet sizes, together with a change in density. *Sglt1^−/−^* mice harbored a 60% higher number of cells per islet compared to healthy pancreatic islets due to decreased cellular apoptosis. In addition, most of the *Sglt1*^−/−^ islets displayed an atypical cytomorphology characterized by a decrease in β-cells and hyperplasia of α-cells (Mühlemann *et al.* 2018). It is well known that α-cells can transdifferentiate into β-cells under certain circumstances (Collombat *et al.* 2009, Thorel *et al.* 2010, Habener & Stanojevic 2012). It has been shown that transdifferentiation of α- to β-cells can be mediated by GLP-1, as GLP-1 can induce the expression of β-cell-specific transcription factors, such as PDX1, in α-cells (Lee *et al.* 2018, Zhang *et al.* 2019). This process involves dedifferentiation of mature α-cells into less differentiated pro-α cells. These cells transition from producing glucagon, which is involved in glucose metabolism, to an undifferentiated pro-α-cell state, where they provide local growth factors, such as GLP-1, that contribute to the generation of β-cells (Habener & Stanojevic 2012, Fava *et al.* 2016). In addition, several studies have highlighted that during the process of transdifferentiation, the α-cells develop hyperplasia, followed by increased plasma levels of GLP-1, and an increased production of GLP-1 in the islets (Gelling *et al.* 2003, Sloop *et al.* 2004, Liu *et al.* 2011, Whalley *et al.* 2011). These findings appear in contrast to our patient, who harbored a tumor with the immunohistochemical profile of a β-cell lineage and without any signs of α-cell differentiation (PDX1+/ARX−), although the exact consequences of *SLGT1* knockout on α-β cell communication need to be fully clarified. It is important to acknowledge the limitations of this report. First, as a single case study, the findings may not be generalizable to other patients with insulinoma and CGGM. Second, the lack of functional studies investigating the molecular mechanisms underlying the coexistence of these two conditions limits the ability to draw definitive conclusions about their relationship. Further research, including functional assays and studies with larger patient cohorts, is necessary to better understand the pathophysiology and potential clinical implications. In conclusion, we uncover a novel association between CGGM and insulinoma. To our knowledge, this is the first description of a patient with a germline *SLC5A1* mutation that developed an insulinoma. Besides the aberrant CNV profile, we hypothesize that SGLT-1 function could play a role in determining and regulating pancreatic islet cytoarchitecture, and in controlling cellular proliferation and apoptosis. Another potential pathogenic cause could result from increased β-cell stimulation by GLP-1 and GIP. Further research would be needed to clarify this relationship and whether CGGM patients truly are at risk of developing insulinoma in adulthood.

## Declaration of interest

AP, JK, AMvdW, MFvV, RTPvC, and LAAB have no potential conflict of interest. JH has received speaker or consultancy fees from Novartis, Ipsen, and Serb. WWdH has received travel fees from Recordati and served on the Advisory Boards of Novartis/Adacap, Camurus, and Ipsen.

## Funding

This work was supported by the Erasmus MC Foundation.

## Author contribution statement

All authors made individual contributions to authorship. JH, WWdH, and AMvdW were involved in the diagnosis and management of this patient. AP and JK were involved in the manuscript submission. MFvV, RTPvC, and LAAB were involved in pathological examination and DNA analysis. All authors reviewed and approved the final draft.

## Patient consent

The patient gave written informed consent for all genetic analyses, histopathological examinations, and publication of the results. Clinical, hormonal, and imaging data were obtained from the electronic patient file.
